# The immune suppressive tumor microenvironment in multiple myeloma: The contribution of myeloid-derived suppressor cells

**DOI:** 10.3389/fimmu.2022.1102471

**Published:** 2023-01-16

**Authors:** Claudia Giannotta, Federica Autino, Massimo Massaia

**Affiliations:** ^1^ Laboratorio di Immunologia dei Tumori del Sangue (LITS), Centro Interdipartimentale di Biotecnologie Molecolari “Guido Tarone”, Dipartimento di Biotecnologie Molecolari e Scienze della Salute, Università degli Studi di Torino, Torino, Italy; ^2^ SC Ematologia, AO S.Croce e Carle, Cuneo, Italy

**Keywords:** MDSC (myeloid-derived suppressor cell), TME (tumor microenvironment), multiple myeloma, Immunothearpies, immune suppression

## Abstract

Myeloid derived suppressors cells (MDSC) play major roles in regulating immune homeostasis and immune responses in many conditions, including cancer. MDSC interact with cancer cells within the tumor microenvironment (TME) with direct and indirect mechanisms: production of soluble factors and cytokines, expression of surface inhibitory molecules, metabolic rewiring and exosome release. The two-way relationship between MDSC and tumor cells results in immune evasion and cancer outgrowth. In multiple myeloma (MM), MDSC play a major role in creating protumoral TME conditions. In this minireview, we will discuss the interplay between MDSC and MM TME and the possible strategies to target MDSC.

## Introduction

Multiple myeloma (MM) is a paradigm disease in which progression is fueled by intrinsic alterations of myeloma cells and tumor-host interactions in the tumor microenvironment (TME) (1). Disease evolution from monoclonal gammopathy of undetermined significance (MGUS) to smoldering myeloma (SMM), and symptomatic disease is characterized by a progressive increase of myeloma cells associated with co-evolving immunological and metabolic changes making the TME unable to hold the disease in check (1). We and others have shown that immune alterations are already detectable in the very early stage of the disease (2, 3) and that they persist in the remission phase (2). The immune MM TME contexture consists of effector cells (i.e, conventional T cells, unconventional T cells like NKT cells, γδ T cells, NK cells etc), professional suppressor cells [i.e, regulatory T cells (Tregs), regulatory B cells (Bregs), myeloid derived suppressor cells (MDSC)], and cells that are functionally conditioned by the TME and acquire protumoral functions like bone marrow stromal cells (BMSC), endothelial cells, osteoblasts (OB), and osteoclasts (4). Recently, BM-resident neutrophils have also been reported to contribute to the TME-induced suppressive commitment of MM patients (5). Unbalanced distribution of effector and suppressor cells already detectable in MGUS is induced by the progressive accumulation of myeloma cells driven by genetic and epigenetic drivers. The bone marrow (BM), which is where MM originates and propagates, has the capacity to physiologically host around 2-5% polyclonal plasma cells. When myeloma cell infiltration overcomes this threshold, the TME is immunologically and metabolically shaped to support myeloma cell growth, to induce drug resistance, and to suppress immune recognition. MDSC play a major role in the protumoral reset of MM TME.

We have previously shown that MDSC are significantly increased in the BM of MGUS and MM patients: granulocytic/polymorphonuclear MDSC (PMN-MDSC), and not monocytic MDSC (M-MDSC), are responsible for the increase (2). MDSC frequency is very similar in MGUS, MM at diagnosis, and MM in relapse. Unexpectedly, we have found that MDSC frequency is significantly higher in MM in remission (2), indicating that there is no correlation between the proportion of BM myeloma cells and MDSC expansion. Similar data have been reported in mouse models in which MDSC start to accumulate in the TME as early as one week after tumor inoculation when the frequency of myeloma cells is very low (<10%) as in MGUS individuals (6).

Approximately, 20-40% of MDCS express the Programmed Cell Death-Ligand 1+ (PD-L1+) (2) and therefore are very well-suited to engage and suppress immune effector cells like Vγ9Vδ2 cells and NK cells expressing the Programmed Cell Death-1 (PD-1) receptor (2). MDSC are PD-L1+ in MGUS and MM irrespective of the disease stage, including MM in remission when most myeloma cells have been cleared from BM (2). The persistence of PD-L1+ MDSC can hinder the immunomodulatory activity of drugs like bortezomib or lenalidomide after autologous stem cell transplantation.

In conclusion, MDSC play a major role in the establishment of the immune suppressive TME in MM. The aim of this minireview is to discuss the mechanisms exploited by MDSC in cooperation with myeloma cells, professional immune suppressor cells, and other bystander cells to promote myeloma cell growth in the BM of MM patients. We will also discuss possible interventions to dampen the immune suppression operated by MDSC and other suppressor cells to recover the antimyeloma activity of immune effector cells.

## MDSC subsets and differentiation

MDSC play a major role in the regulation of immune homeostasis in healthy individuals, and the regulation of immune responses in infectious diseases, autoimmunity, aging, pregnancy, transplantation, and obesity (7). In cancer, the immune suppressive activity of MDSC is exploited by tumor cells to evade immune surveillance and support their survival and accumulation (7).

MDSC are derived from bone marrow hematopoietic stem cells (7). There are two major subsets of MDSC in humans: PMN-MDSC and M-MDSC. The first one are phenotypically and morphologically similar to neutrophils (CD15^+^ and/or CD66b^+^), whereas M-MDSC are similar to monocytes (CD14+)(7). More recently, a third subset of phenotypically distinct immature early-MDSC (e-MDSC) has been identified in cancer patients (8). In this review we will use the term MDSC to identify both PMN-MDSC and M-MDSC unless otherwise specified.

MDSC development occurs in two partially overlapping waves (9). The first one is driven by cytokines and soluble factors including granulocyte-macrophage colony-stimulating factor (GM-CSF), macrophage colony-stimulating factor (M-CSF), granulocyte colony-stimulating factor (G-CSF), interleukin 6 (IL-6), and vascular endothelial growth factor (VEGF). These cytokines and soluble factors are produced by tumor cells and/or BMSC in the TME and promote MDSC differentiation from hematopoietic progenitor cells *via* STAT3 and STAT5 activation (10, 11, 12). Mesenchymal stromal cells (MSC) also induce MDSC expansion *via* the hepatocyte growth factor (HGF), c-Met, and STAT3 phosphorylation (10). The second wave is driven by a different set of cytokines and inflammatory soluble factors like interleukin 13 (IL-13), toll-like receptor (TLR) ligands, and prostaglandin E2 (PGE2) yielding to the functional MDSC activation *via* the STAT1 and NF-kB pathways (10–12). The TME is highly predisposed to drive the expansion and activation of MDSC at the expense of other myeloid-derived cells like monocytes, macrophages and dendritic cells (DC) (8).

## Immuno suppressive MDSC features

The immune suppressive MDSC activity is dependent on: 1) the depletion of essential CD8^+^ T- cell nutrients in the TME; 2) the production of immune suppressive cytokines and/or soluble factors; 3) the expression of cell surface inhibitory molecules [i.e., (PD-L1)]; 4) the protumoral metabolic TME rewiring at the expense of immune effector cells.

### Amino acid depletion

MDSC express the xc- transporter and import cystine, but, unlike DC and macrophages, they are unable to export cysteine because they lack the ASC neutral amino acid transporter (13). Considering the progressive TME invasion by tumor cells and MDSC at the expense of other cells which can supply extracellular cysteine, the TME becomes depleted of cysteine jeopardizing the activation of CD8+T cells that are unable to convert cystine to cysteine to meet their metabolic requirements (13).

MDSC also deplete the TME of tryptophan *via* the enzyme indoleamine 2, 3-dioxygenase (IDO) (14). T lymphocytes are very susceptible to tryptophan shortage which restrains their proliferative responses by inducing an integrated stress response and the inactivation of the mTOR pathway (15, 16). Tryptophan catabolites can also induce the apoptosis of cytotoxic T cells (17, 18), and the concurrent differentiation of Tregs (16). L-arginine (L-arg) is another essential amino acid which is critical for T-cell immune functions. Arginine metabolism is regulated by the inducible nitric oxide synthase (iNOS) isoenzymes, arginase (Arg 1/2) activity, and proline and polyamines synthesis. MDSC express both iNOS and Arg-1 that induce L-arg depletion in the TME leading to inhibition of CD3-ζ expression in T cells, and induction of apoptosis (7, 9, 19).

### Cytokines and soluble factors

The production and release of suppressor cytokines and soluble factors is another mechanism exploited by MDSC to protect tumor cells from immune recognition and killing. Nitric oxide (NO), reactive oxygen species (ROS), peroxynitrite (PNT) (a short-lived product of NO reaction with ROS), interleukin 10 (IL-10), and transforming growth factor-β (TGF-β) are released by MDSC with slightly difference between PMN-MDSC and M-MDSC subsets (7, 9, 20, 21). The hyper-production of ROS and PNT in the TME impairs the ability of CD8+ T cells to bind to peptide–major histocompatibility complexes and to respond to specific peptides (21). NO also hampers the Fc receptor-mediated effector functions of NK cells (22). IL-10 recruits Tregs in the TME and decreases CD8+ T-cell antigen sensitivity by inducing cell surface glycoprotein branching (23). TGF-β is induced by IL-13 (24) and interferon-γ (IFN-γ) (25), and contributes to T-cell suppression through Tregs development (25). Kynurenine is another soluble immune suppressive factor that is generated in the TME as a consequence of tryptophan catabolism by MDSC. Kynurenine can inhibit T-cell and NK cell proliferation and drive the differentiation of naïve T cells into Tregs (16).

### Cell surface molecules

The cell surface expression of immune checkpoints ligands (ICP-L) like PD-L1 is another mechanism used by M-MDSC to suppress immune effector cells (2, 7, 9), while PMN-MDSC preferentially exploit the Fas/Fas-ligand pathway to induce T-cell depletion in the TME (26). The V-domain immunoglobulin suppressor of T cell activation (VISTA) is a novel co-inhibitory ligand/receptor highly expressed by MDSC in the TME that suppresses T-cell effector functions and contributes to acquired resistance to PD-1/PD-L1 blockade (27). Lastly, CXCR2 is another cell surface molecule that is critical in mice models and paediatric sarcoma to promote the accumulation of MDSC in the TME and hamper the efficacy of anti-PD-1 treatment (28).

### Protumoral metabolic TME rewiring

The TME is a very dynamic ecosystem that is progressively molded by tumor cells to locally create protective conditions to support their growth and resistance to therapy, from conventional chemotherapy to immunotherapy (29, 30). Hypoxia is a major metabolic feature of TME (30), especially in solid tumors, almost always associated with the extracellular acidification induced by lactate accumulation. Tumor cells rewire their metabolism to survive and proliferate in the TME by: 1) increasing glucose and amino acid uptake, glycolytic flux, and lactate production; 2) modifying glutamine metabolism, tricarboxylic acid cycle, and oxidative phosphorylation; 3) increasing the production of mitochondrial ROS; 4) modulating fatty acid synthesis and oxidation (FAO) (30). MDSC partially mimick the metabolic rewiring of tumor cells by adapting their lactate, glucose, and lipid metabolism to the hypoxic and acidic TME conditions (31, 32). As a result, MDSC survive in the TME, contribute to the exacerbation of the protumoral metabolic TME commitment, and maintain unaltered their immune suppressor activity (33–35).

## Immune suppressive and metabolic features in MM

MM is a hematologic cancer characterized by the accumulation of malignant plasma cells (myeloma cells) in the BM. Progressive colonization of BM results in a deep remodelling of the BM niche that becomes committed to support myeloma cell growth, immune evasion, and drug resistance (1).

MDSC play a major role in establishing the protumoral TME commitment. We have shown that MDSC accumulation in the BM is already detectable in MGUS, and their expansion persists throughout the entire period of the disease (2), including the remission phase (2). In our hands, PMN-MDSC was the main subpopulation to be expanded in MGUS and MM (2), while other groups have reported the predominance of M-MDSC in MM at diagnosis and in relapse (36, 37). Immunogenomic characterization identified CD11b+CD13+CD16+ cells as the PMN-MDSC subset with strongest capacity to suppress anti-myeloma activity T-cell immune responses (38). MDSC-like suppressive activity is also exhibited by MM neutrophils (5), suggesting that an accurate characterization of MDSC should be based on phenotypic markers, immunosuppressive potential, and transcriptional network.

Development and suppressor functions of MDSC are supported by myeloma cells and bystander cells *via* direct and indirect mechanisms. Direct mechanisms operated by myeloma cells include: 1) IL-6 production (39, 40) which prevents MDSC differentiation and promotes MDSC accumulation and activation *via* the STAT3 signaling pathway (41); 2) the induction of Mcl-1, an anti-apoptotic protein sustaining MDSC survival (42); 3) the secretion of galectin-1 that targets CD304 on MDSC and enhances their immune suppressive capacity (43); 4) the production of chemokine ligand 5 (CCL5) and macrophage migration inhibitory factor (MIF) (44). MIF has also been reported to potentiate the immune suppressive activity of MDSC *via* CD84-mediated PD-L1 upregulation (45); 5) the release of exosomes that promotes MDSC growth and NO production (46)

Bystander cells in the TME also cooperate with myeloma cells in the induction and activation of immune suppressive MDSC *via* direct mechanisms including: 1) IL-6 release (47, 48); 2) exosome release by BMSC (49); 3) production and release of immune suppressive molecules [i.e. Prostaglandin-Endoperoxide Synthase 2 (PTGS2), TGF-β, Nitric Oxide Synthase 2 (NOS2), IL-10 and IL-6] by MSC and OB (50, 51).

In addition to the direct mechanisms listed above, myeloma cells and bystander cells promote the accumulation and activation of MDSC *via* indirect mechanisms. An example is the metabolic rewiring operated by myeloma cells and bystander cells that creates an hypoxic and nutrient-depleted TME that promotes the accumulation and activation of MDSC at the expense of immune effector cells (52–54). Lactate over-production shifts MDSC differentiation toward PMN-MDSC (55), which is the subset that we and others have shown to be increased in the peripheral blood (PB) and BM of MM patients (2, 56).

The accumulation and activation of MDSC is beneficial to myeloma cells creating a very effective protumoral loop (3, 57). MDSC facilitate the self-renewal of myeloma stem-cells, enhance their tumorigenic potential *via* epigenetic regulation (58), and promote myeloma cell survival *via* AMPK phosphorylation leading to increase β-oxidation, ATP production, and increased NADPH levels (59). MDSC production of S100A9, a calcium-binding protein that promotes the release of TNF-α, IL-6, and IL-10 in autocrine pathway through TLR4 interaction, attracts myeloma cells in the TME (60) and supports myeloma cell growth *via* the activation of the canonical NFκB pathway (61).

Indirect mechanisms operated by MDSC to support myeloma cells are deprivation of nutrients, production of soluble factors, and the expression of cell surface inhibitory molecules. The common denominator is the impairment of anti-myeloma immune responses. In addition, PMN-MDSC are educated to express angiogenesis-related proteins to support tumor angiogenesis (62).

MDSC upregulate enzymes that contribute to the shortage of amino acids essential for immune effector T cells. Arginase 1 (Arg-1) expression and NO production by MDSC limit the availability of L-Arg needed for effective TCR-mediated signaling (63, 64). MDSC can utilize glutamine for anaplerosis like myeloma cells (65, 66), exacerbating glutamine deprivation in the TME (54).

Several soluble factors and cytokines contribute to the immune suppressor activity of MDSC in the TME, like IL-10, IL-6, TGF-β, CD40-CD40 Ligand, and IFN-γ. These cytokines tip the scales in favor of Tregs (44, 67), whose number is directly correlated with MDSC expansion (56). Lastly, CD38 expression on MDSC (68) contributes to the discontinuous multicellular pathway of adenosine (Ado), an immune suppressive nucleoside highly represented in the TME of MM patients (69).

The expression of immune checkpoint (ICP)/ICP-L contributes to the impairment of anti-myeloma immune responses. We have previously demonstrated that PD-L1 is expressed by BM MDSC in all disease states (2) and can contribute to hold in check anti-myeloma activity of PD1+ effector cells such as T cells, NK cells, and Vγ9Vδ2 T cells. Recently, it has been reported in solid tumors that MDSC can boost the immune suppressive activity of Bregs against T cells *via* the PD-1/PD-L1 axis (70, 71).

Lastly, MDSC can trans-differentiate into functional osteoclasts (72) to combine immune suppressive functions and direct protumoral functions (73). In mice models, G-MDSC have also been shown to promote angiogenesis (62), another major protumoral TME disruption occurring in human MM (62).

**Figure 1:**
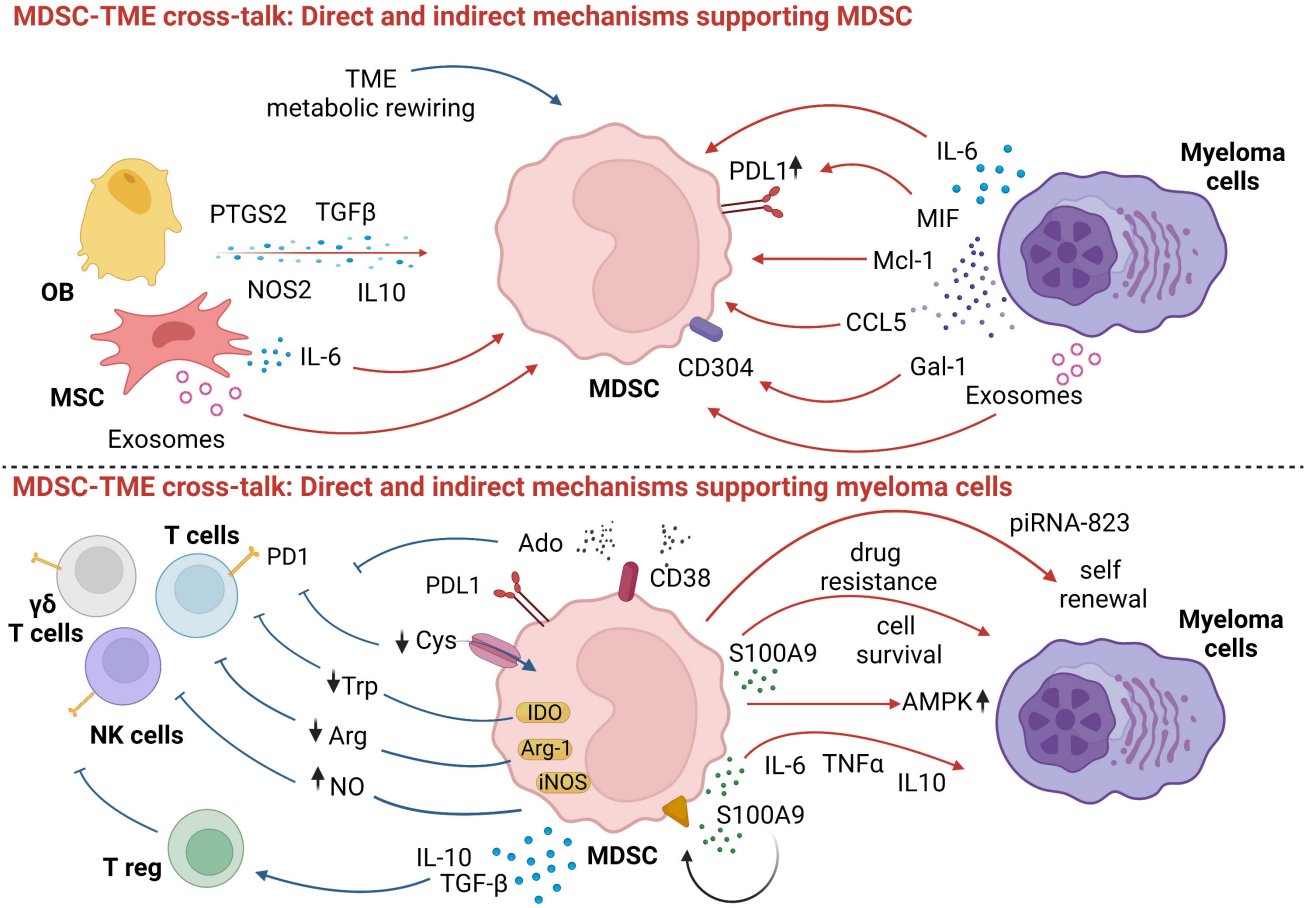
Myeloma cell and surrounding cells promote MDSC differentiation and suppressive functions. In turn, MDSC undermine anti-tumor immune responses and guarantee myeloma cells survival and growth. Red arrows: direct mechanisms; blue arrows: indirect mechanisms. Created by BioRender.com.

The direct and indirect mechanisms involved in the cross-talk between MDSC, myeloma cells, immune effector, immune suppressor cells, and other bystander cells in the TME of MM patients are shown in **Figure 1**.

## Therapeutic interventions

The correlation between the frequency of MDSC and the clinical outcome identifies these cells as potential targets of immune-based therapeutic interventions (74). However, the therapeutic targeting of MDSC is not easy given their multifaceted biological functions and multiple interactions in the TME. Possible strategies are: 1) to restrain their accumulation in the PB and TME; 2) to prevent their functional activation in the TME; 3) to block their protumoral interactions with myeloma cells and bystander cells.

MDSC accumulation can be restrained by immunomodulatory drugs (IMiDs) (44) and proteasome inhibitors (PI) (59). A cereblon (CRBN)-dependent and -independent down-regulation of CCL5 and MIF is a possible mechanism of IMiDs activity on MDSC (44) that can be improved by Arg-1 inhibitors (75). Clinical data confirm the capacity of IMiDs to restrain MDSC *in vivo* as shown by the decrease of PB MDSC in MM patients treated with pomalidomide, dexamethasone, and daratumumab (76). Daratumumab can also exert a favourable immune modulatory activity in the TME of MM patients by depleting CD38+ MDSC *via* antibody-dependent cellular cytotoxicity (ADCC) and complement-dependent cytoxicity (CDC) (68). Data from mice models indicate that demethylating agents like decitabine (DAC), IL-18, and zoledronic acid (ZA) can also affect MDSC survival in the TME (72, 77, 78). ZA is currently used in MM and other solid cancers to prevent osteoclast activation and bone lesions, but this molecule is also endowed with pleiotropic immune modulatory activity (79), including the capacity in murine models to reduce the numbers of MDSC and prevent their differentiation into osteoclasts (72). Targeting CD84 and the CD304-Gal1 axis are other strategies used in myeloma mouse models to restore anti-myeloma T-cell responses by reducing MDSC accumulation and PD-L1 expression (45).

The immune suppressive activity of MM MDSC has also been inhibited *in vitro* using ABR-238901, a small molecule inhibiting S100A9 interactions (60), and tasquinimod (74). Anti-estrogen therapy may also restrain MDSC suppressive activity, since 17β-estradiol increases their ability to suppress T-cell proliferation (80). iNOS and Arg-1 activities have been down-modulated in mice models with tadalafil (81), a PDE5 inhibitor that has been used with some evidence of clinical efficacy in relapsed/refractory MM patients in combination with lenalidomide (82). Protumoral MDSC cellular interactions in the TME can also be limited by interrupting ICP/ICP-L interactions (2). Daratumumab in combination with the anti-PD1 monoclonal antibody cetrelimab has been reported to decrease the number of circulating MDSC and increase that of CD8+ T cells in the PB of MM patients in relapse (83). In acute myeloid leukemia (AML), knockdown of VISTA, a negative checkpoint regulator in the B7 family, reduced the MDSC-mediated inhibition of T cells (84). Data are not available in MM yet, but VISTA up-regulation is also expected in the BM of MM given the hypoxia and low pH as reported in solid cancer (85).

In conclusion, understanding the mechanisms underlying the immune suppressive activity of MDSC in MM will pave the ground to the therapeutic targeting of these cells to improve the efficacy of immune-based treatments in MM.

## Author contributions

CG, FA and MM contributed to the writing of the manuscript, CG designed the figure, MM revised the manuscript.
